# The C-terminal portion of the cleaved HT motif is necessary and sufficient to mediate export of proteins from the malaria parasite into its host cell

**DOI:** 10.1111/mmi.12133

**Published:** 2013-01-21

**Authors:** Sarah J Tarr, Adam Cryar, Konstantinos Thalassinos, Kasturi Haldar, Andrew R Osborne

**Affiliations:** 1 Institute of Structural and Molecular Biology, Division of Biosciences, Birkbeck and University College LondonLondon, UK; 2 Department of Biological Sciences, Center for Rare and Neglected Diseases, University of Notre DameNotre Dame, IN, 46556, USA

## Abstract

The malaria parasite exports proteins across its plasma membrane and a surrounding parasitophorous vacuole membrane, into its host erythrocyte. Most exported proteins contain a Host Targeting motif (HT motif) that targets them for export. In the parasite secretory pathway, the HT motif is cleaved by the protease plasmepsin V, but the role of the newly generated N-terminal sequence in protein export is unclear. Using a model protein that is cleaved by an exogenous viral protease, we show that the new N-terminal sequence, normally generated by plasmepsin V cleavage, is sufficient to target a protein for export, and that cleavage by plasmepsin V is not coupled directly to the transfer of a protein to the next component in the export pathway. Mutation of the fourth and fifth positions of the HT motif, as well as amino acids further downstream, block or affect the efficiency of protein export indicating that this region is necessary for efficient export. We also show that the fifth position of the HT motif is important for plasmepsin V cleavage. Our results indicate that plasmepsin V cleavage is required to generate a new N-terminal sequence that is necessary and sufficient to mediate protein export by the malaria parasite.

## Introduction

According to the World Health Organization, there were approximately 781 000 malaria-related deaths in 2009 (W.H.O., 2009). The disease is caused by unicellular eukaryotes, such as *Plasmodium falciparum*. The parasite life cycle is complex, but symptoms of the disease occur when the parasite replicates inside erythrocytes. Within the erythrocyte, the parasite is contained within a vacuole, referred to as the parasitophorous vacuole (PV). By exporting proteins across its plasma membrane, the surrounding PV membrane, and into the erythrocyte, the parasite alters properties of its host cell (Haldar and Mohandas, 2007; Goldberg and Cowman, 2010). The mechanism of protein export is poorly understood, but many exported proteins are essential for parasite survival (Maier *et al*., 2008) and virulence (Kraemer and Smith, 2006; Maier *et al*., 2009).

The first step in the export of most proteins is targeting to the parasite endoplasmic reticulum, mediated by an N-terminal transmembrane domain (Knuepfer *et al*., 2005c; Boddey *et al*., 2010). An additional sequence, referred to as a Host Targeting (HT) motif (also referred to as a PEXEL), is also required for proteins to be transported across the PV membrane (Hiller *et al*., 2004; Marti *et al*., 2004; Przyborski *et al*., 2005). The HT motif is located approximately 35 residues downstream of the N-terminal transmembrane domain and corresponds to the consensus sequence RxLxE/D/Q, (x is any amino acid) (Hiller *et al*., 2004; Marti *et al*., 2004; Przyborski *et al*., 2005; Sargeant *et al*., 2006; van Ooij *et al*., 2008). Prior to export, the HT motif is proteolytically cleaved after the Leu residue to release a C-terminal fragment with a new N-terminal sequence that starts xE/D/Q (Chang *et al*., 2008; Boddey *et al*., 2009). Cleavage occurs in the parasite endoplasmic reticulum (Chang *et al*., 2008; Osborne *et al*., 2010) and is mediated by the protease plasmepsin V (Boddey *et al*., 2010; Russo *et al*., 2010). The new N-terminus is N-acetylated by an unknown enzyme (Chang *et al*., 2008). Cleaved and N-acetylated proteins are trafficked to the PV where they are unfolded and translocated across the PV membrane (Gehde *et al*., 2009). It is thought that the latter step is mediated by the PTEX complex that includes an ATPase and a putative protein conducting channel located in the PV membrane (de Koning-Ward *et al*., 2009).

Several models have been put forward to explain the contribution of the HT motif to protein export (Boddey *et al*., 2010; Bhattacharjee *et al*., 2012b). Mutation of the Arg or Leu residues to Ala blocks protein export (Hiller *et al*., 2004; Marti *et al*., 2004; Przyborski *et al*., 2005; Boddey *et al*., 2009). Mutation of the Glu/Asp/Gln residue to Ala either has little affect on export (Grüring *et al*., 2012; Bhattacharjee *et al*., 2012a) or partially blocks export (Boddey *et al*., 2009). It has been proposed that the information required for export resides predominantly in the RxL sequence and that targeting of a protein for export occurs before cleavage of the HT motif, or is directly coupled to the cleavage event. Consistent with this a previous study showed that a protein whose N-terminus is generated by signal peptidase and resembles the released C-terminal fragment of the cleaved HT motif, starting xE/D/Q, is not exported (Boddey *et al*., 2010). Hence, it has been proposed that cleavage by plasmepsin V may be directly coupled to an obligatory handoff to the next component in the export pathway (Boddey *et al*., 2010).

A receptor for the uncleaved HT motif has not been identified. However, the motif binds to phosphatidylinositol-3-phosphate (Bhattacharjee *et al*., 2012b). It is thought that sorting in the parasite ER is mediated by the interaction of the intact HT motif with PI(3)P on the luminal side of the ER membrane (Bhattacharjee *et al*., 2012b). Nonetheless, protein chimeras containing only the C-terminal fragment of the cleaved HT motif were exported but this was proposed to be via an HT-motif independent export pathway (Bhattacharjee *et al*., 2012b) that can act in conjunction with the HT signal (Bhattacharjee *et al*., 2012a).

We propose a model in which the HT motif is cleaved by plasmepsin V and that the newly generated N-terminal sequence starting xE/D/Q is recognized late in the secretory pathway and is sufficient to mediate export. We show that a protein targeted to the ER lumen, lacking the RxL portion of the HT motif but whose N-terminus starts with the sequence xE/D/Q, is efficiently exported by the parasite. Notably, we bypass the requirement for the RxL portion of the HT motif, and plasmepsin V cleavage, by using an exogenous protease to generate an N-terminal xE/D/Q sequence in an exported protein. This shows that the role of plasmepsin V is to cleave the HT motif and reveal a new N-terminal sequence that is sufficient to mediate export. Using multiple HT motif-containing model proteins, we show that the identity of the amino acids in the fourth and fifth positions of the HT motif, as well as amino acids further downstream, influence the efficiency of protein export. Importantly, these mutations behave in a similar manner in proteins whose new N-terminal xE/D/Q sequence is generated by plasmepsin V or an exogenous protease indicating that these proteins are exported by the same export pathway. Together these data show that the role of plasmepsin V cleavage is to generate the C-terminal fragment of the cleaved HT motif that is both necessary and sufficient to target a protein for export.

## Results

### The C-terminal fragment of the cleaved HT motif is sufficient to mediate protein export

**Fig. 1:**
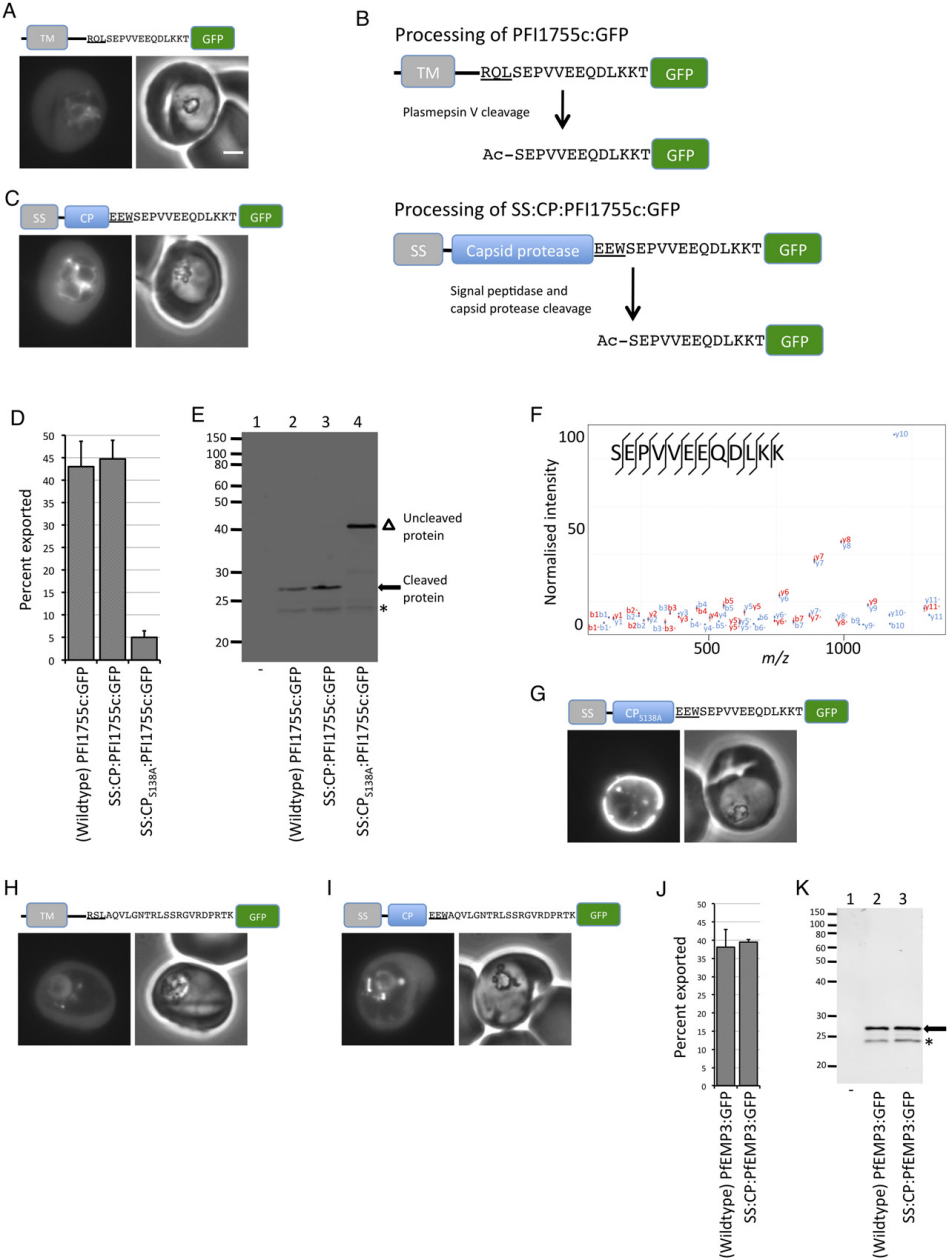
Cleavage of the HT motifs in PFI1755c and PfEMP3 releases a C-terminal fragment that is sufficient to mediate protein export. A. Images of a parasite expressing wild-type PFI1755c_1–61_:GFP. The left- and right-hand images show GFP localization and a phase contrast image respectively. A diagram illustrating the construct type is also shown. The amino acid sequence of the protein adjacent to the protease cleavage site is written using the single letter amino acid code. The three residues preceding the protease cleavage site are underlined. TM, transmembrane segment; GFP, green fluorescent protein. Scale bar, 2 μm. B. Diagram illustrating the proteolytic cleavage of PFI1755c_1–61_:GFP by plasmepsin V and the self-cleavage of SS:CapProt:PFI1755c_49–61_:GFP. Illustrations are as in Fig. 1A. SS, signal sequence. C. Images of a parasite expressing SS:CapProt:PFI1755c_49–61_:GFP. The left- and right-hand images show GFP localization and a phase contrast image respectively. A diagram illustrating the construct type is also shown (as for Fig. 1A). CP, capsid protease. D. Quantification of GFP export of PFI1755c_1–61_:GFP and SS:CapProt:PFI1755c_49–61_:GFP constructs. The percentage of GFP fluorescence localized in the erythrocyte is shown. The GFP-tagged proteins expressed in each parasite are indicated. Error bars show standard deviation. E. Anti-GFP Western blot of parasites expressing PFI1755c_1–61_:GFP, SS:CapProt:PFI1755c_49–61_:GFP or a similar construct in which the active site of the capsid protease is mutated (Ser138 to Ala). The constructs are indicated below the blot. Triangle: SS:CapProt:PFI1755c_49–61_:GFP that has not been processed by the capsid protease. Closed arrow: protein that has been processed by either plasmepsin V or capsid protease. Asterisk: GFP degradation product. Lane 1 contains material from untransfected parasites. F. Averaged tandem mass spectrum for the peptide N-acetyl-SEPVVEEQDLKK, derived from PFI1755c_1–61_:GFP. Mean b and y ion intensities for two experiments are in blue and red respectively. Standard deviation of triplicate measurements for each ion intensity, normalized to the most abundant ion, is shown. *, neutral loss of ammonia; ∧, neutral loss of water. G. Images of a parasite expressing SS:CapProt:PFI1755c_49–61_:GFP in which the capsid protease domain contains the S138A active site mutation. H–I. Images of parasites expressing GFP-tagged PfEMP3. GFP image panels, phase contrast panels and illustrations are arranged as in Fig. 1A. Wild-type PfEMP3_1–82_:GFP (H); SS:CapProt:PfEMP3_63–82_:GFP (I). J. Quantification of GFP export of PfEMP3 constructs. The percentage of GFP fluorescence localized in the erythrocyte is shown. The GFP-tagged proteins expressed in each parasite are indicated. Error bars show the standard deviation. K. Anti-GFP Western blot of PfEMP3_1–82_:GFP and SS:CapProt:PfEMP3_63–82_:GFP; labelled as in (E). Lane 1 contains material from untransfected parasites.

We tested whether the C-terminal portion of the cleaved HT motif is sufficient to mediate export of PFI1755c (REX3) (Spielmann *et al*., 2006), which has the HT motif ^46^RQLSE^50^. A protein comprising residues 1–61 of PFI1755c fused to GFP (PFI1755c_1–61_:GFP) (Sargeant *et al*., 2006) was efficiently exported to the host cell (Fig. 1A). Cleavage of the HT motif generates a new N-terminal sequence starting with Ser and Glu as the first and second residues respectively. By convention, the two residues located on the C-terminal side of a protease cleavage site are referred to as P1′ and P2′ residues; the P1′ residue is one amino acid from the cleavage site and the P2′ residue is two amino acids from the cleavage site (Schechter and Berger, 1967); hereafter we use this convention.

To test whether the C-terminal fragment of the cleaved HT motif is sufficient to mediate export, we exploited the protease domain from the Semliki forest virus capsid protein. This domain is a self-cleaving protease; once the protease folds, its C-terminus loops into the active site and is cleaved in *cis* after a specific Trp residue (Choi *et al*., 1997; Kowarik *et al*., 2002). The region following this Trp residue is released from the protein. To test whether the cleaved HT motif is sufficient to mediate protein export, we generated a fusion protein comprising an ER-targeting signal sequence, followed by the capsid protease domain, residues 49–61 of PFI1755c, and a C-terminal GFP tag (SS:CapProt:PFI1755c_49–61_:GFP; Fig. 1B). Self-cleavage by the capsid protease will release a C-terminal fragment that starts with the N-terminal P1′ P2′ sequence, Ser Glu, which is identical to the N-terminus of PFI1755c_1–61_:GFP generated by plasmepsin V cleavage (Fig. 1B).

SS:CapProt:PFI1755c_49–61_:GFP was expressed in *P. falciparum.* Consistent with the idea that the new N-terminus generated by cleavage of the HT motif is sufficient to mediate protein export, the GFP protein was efficiently exported into the host erythrocyte (Fig. 1C). Quantification of the GFP fluorescence localized in the erythrocyte cytoplasm showed that export efficiency was similar in parasites expressing SS:CapProt:PFI1755c_49–61_:GFP and PFI1755c_1–61_:GFP (Fig. 1D). Analysis by Western blotting showed that a GFP-tagged fragment was generated by capsid protease cleavage, the size of which matched that generated by plasmepsin V mediated cleavage of PFI1755c_1–61_:GFP (Fig. 1E, closed arrow, compare lanes 2 and 3). Mass spectrometry analysis of tryptic peptides derived from immunoprecipitated SS:CapProt:PFI1755c_49–61_:GFP and PFI1755c_1–61_:GFP indicated that these proteins indeed have identical P1′ P2′ N-terminal sequences. Although the N-termini are not completely homogeneous, the peptide derived from the cleaved and N-acetylated HT motif, N-Ac-SEPVVEEQDK(K), was the most N-terminal peptide identified in both samples (Table 1 and Fig. 1F).

**Table 1:** Analysis of protein N-terminal sequences by mass spectrometry

			Experiment 1			Experiment 2		
				
Sample (mutation)	N-terminal peptides	Acetylation	Error (ppm)	Error SD	Percent matched products	Error (ppm)	Error SD	Percent matched products
PFI1755c_1–61_:GFP (wild-type)	SEPVVEEQDLK	N-acetyl	1.91	1.9	51.5	1.11	2.7	100
	SEPVVEEQDLKK	N-acetyl	0.98	2.23	75	−0.5	1.71	100
	PVVEEQDLK		0.56	2.13	48.1	−0.8	1.76	66.7
PFI1755c_1–61_:GFP (P1′ to Asp)	DEPVVEEQDLK		−0.66	1.37	54.5	−1.86	1.15	54.5
	DEPVVEEQDLKK		2.44	2.67	47.2	0.47	2.32	45.8
	DEPVVEEQDLKK	N-acetyl	0.54	3.12	83.3	−1.39	0.1	54.2
	PVVEEQDLKK		4.29	1.56	90	1.07	2.34	95
PFI1755c_1–61_:GFP (P1′ and P2′ to Tyr–Gly)	YGPVVEEQDLK		1.79	2.31	100	2.74	0.87	100
	YGPVVEEQDLKK		2.8	1.59	100	1.29	1.46	97.2
	PVVEEQDLKK		2.31	0.76	90	1.18	3.1	90
	EEQDLKK		−0.99	0.78	57.1	0.02	2.19	52.4
	VEEQDLKK		1.07	2.5	83.3	−0.53	1.6	70.8
	VVEEQDLKK		1.09	1.86	66.7	−0.38	0.68	63
	GPVVEEQDLKK		1.78	1.51	87.9	1.47	1.54	84.8
SS:CapProt:PFI1755c_49–61_:GFP	SEPVVEEQDLK	N-acetyl	0.67	2.61	87.9	1.37	1.09	63.6
	SEPVVEEQDLKK	N-acetyl	1.3	1.46	97.2	3.69	0.47	83.3
	PVVEEQDLK		0.47	1.12	70.4	0.68	1.5	77.8
SS:CapProt:PFI1755c_49–61_:GFP (P1′ to Asp)	DEPVVEEQDLK		2.56	1.95	84.8	1.53	2.58	78.8
	DEPVVEEQDLKK		2.18	1.14	66.7	2.02	0.09	45.8
	DEPVVEEQDLKK	N-acetyl	2	1.43	52.8	0.16	0.13	50
	PVVEEQDLK		0.61	2.1	77.8	−0.42	0.68	33.3
	PVVEEQDLKK		3.17	2.94	90	3.11	0.5	90
SS:CapProt:PFI1755c_49–61_:GFP (P1′and P2′ to Tyr–Gly)	YGPVVEEQDLK		4.07	2.99	100	3.39	2.05	100
	YGPVVEEQDLKK		0.93	0.83	100	3.99	2.1	100
	PVVEEQDLKK		2.4	2.95	83.3	−0.34	0.93	93.3
	EEQDLKK		−2.23	3.85	52.4	−1.54	2.46	61.9
	VEEQDLKK		0.78	1.46	66.7	1.67	1.53	75
	VVEEQDLKK		1.08	0.73	66.7	−0.34	1.24	63
	GPVVEEQDLKK		1.21	0.81	84.8	1.84	1.59	84.8
SS:CapProt:PFI1755c_49–61_:GFP (P1′ to Ala)	SAPVVEEQDLK	N-acetyl	2.95	3.03	90.9	2.28	3.26	100
	SAPVVEEQDLKK	N-acetyl	0.79	0.71	100	0.5	1.09	95.8
	PVVEEQDLK		1.41	1.93	70.4	1.99	4.65	77.8
	EEQDLK		−2.68	3.12	33.3	−0.84	6.03	50
PFI1755c_1–61_:GFP (sequence downstream of cleavage site: SAPVVAAAALK)	SAPVVAAAALKK	N-acetyl	2.51	1.01	58.3	1.6	2.3	88.9
	VAAAALK		0.77	1.16	64.3	−0.48	0.64	47.6
PFI1755c_1–61_:GFP (sequence downstream of cleavage site: SAPVVSTSTLK)	SAPVVSTSTLK	N-acetyl	1.85	0.95	75.8	−0.91	1.44	63.6
	SAPVVSTSTLKK	N-acetyl	0.55	1.67	86.1	2.37	1.53	77.8

N-terminal peptides derived from the PFI1755c fragment of the indicated proteins are shown from two independent experiments. No peptides N-terminal to the protease cleavage sites of proteins processed by plasmepsin V or capsid protease were identified. GFP-derived peptides are not shown. ppm, parts per million mass accuracy of the precursor ion; percent matched products, percentage of expected b and y ions obtained. The ppm error refers to the standard deviation of three technical replicates within each experiment. Averaged tandem mass spectra for the most N-terminal peptides derived from PFI1755c_1–61_:GFP with the linker region mutated to SAPVVSTSTLKKT, and SS:CapProt:PFI1755c_49–61_:GFP are shown in Fig. S4. SD, standard deviation.

To confirm that the capsid protease does not contain a cryptic export sequence, the active site Ser138 in the capsid protease domain of SS:CapProt:PFI1755c_49–61_:GFP was mutated to Ala (Choi *et al*., 1997). This mutation prevented protease self-cleavage and release of the C-terminal GFP fragment from the protease domain (Fig. 1E, triangle, lane 4). Importantly, the mutation blocked export of GFP to the host cell; GFP accumulated at the periphery of the parasite (Fig. 1G and D). Immunofluorescence analysis showed colocalization of the GFP signal with the PV marker Exp2 (Fig. S1C). These results show that the capsid protease does not contain a cryptic export sequence and that cleavage of SS:CapProt:PFI1755c_49–61_:GFP is mediated by the capsid protease, not by plasmepsin V. Additionally, this indicates that the ability of the P1′, P2′ and following residues to support export is blocked if they are preceded by an N-terminal folded domain, in this case the capsid protease.

We also tested whether the C-terminal portion of the cleaved HT motif of the exported protein *P. falciparum* Erythrocyte Membrane Protein 3 (PfEMP3), was sufficient to mediate protein export. The HT motif of PfEMP3 (^60^RSLAQ^64^) is cleaved by plasmepsin V to yield P1′ P2′ residues, Ala Gln (Boddey *et al*., 2010). A protein comprising residues 1–82 of PfEMP3 fused to GFP (PfEMP3_1–82_:GFP) (Knuepfer *et al*., 2005b) was efficiently exported to the host erythrocyte (Fig. 1H). An ER-targeted capsid protease fused to residues 63–82 of PfEMP3, and tagged C-terminally with GFP (SS:CapProt:PfEMP3_63–82_:GFP) was also exported into the host erythrocyte with similar efficiency (Fig. 1I and J). This shows that the PfEMP3 C-terminal fragment normally generated by HT motif cleavage is sufficient to mediate protein export. Western blotting indicated that both PfEMP3_1–82_:GFP and SS:CapProt:PfEMP3_63–82_:GFP were proteolytically processed as expected (Fig. 1K, closed arrow, lanes 2 and 3 respectively).

Together, these results show that the C-terminal fragments of the cleaved HT motifs of PFI1755c and PfEMP3 are sufficient to mediate efficient protein export.

### The identity of the P1′ and P2′ residues of the HT motif influences the efficiency of protein export and plasmepsin V cleavage

We subsequently tested whether the new N-terminus generated by HT motif cleavage is necessary for protein export. Specifically, we investigated whether mutations in the P1′ and P2′ positions of PFI1755c_1–61_:GFP affected protein export efficiency.

**Fig. 2:**
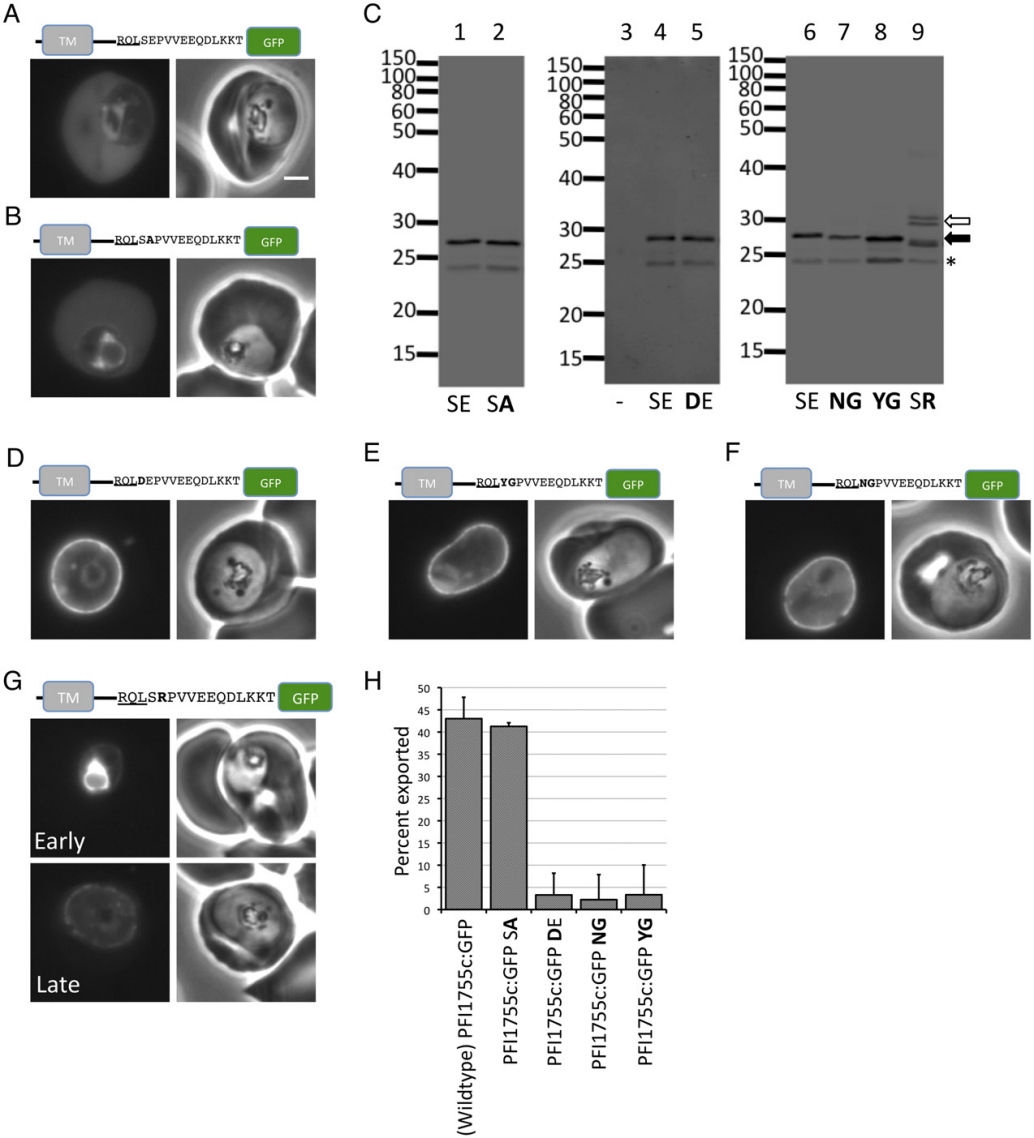
Mutations in the P1′ and P2′ positions in the cleaved HT motif influence the efficiency of protein export. A–B. Localization of PFI1755c_1–61_:GFP proteins. Image panels and illustrations are as in Fig. 1. Scale bar, 2 μm. Wild-type PFI1755c_1–61_:GFP (A); P2′ position is mutated to Ala (B). C. Anti-GFP Western blot of PFI1755c_1–61_:GFP proteins containing mutations in the P1′ and P2′ positions. The amino acid sequence of the P1′ and P2′ positions of each protein is indicated below each lane; mutated residues are in bold. Open arrow: doublet of high molecular weight protein bands that have not been processed by plasmepsin V. Closed arrow: protein that has been processed by plasmepsin V. Asterisk: GFP degradation product. Lane 3 contains material from untransfected parasites. D–F. Localization of PFI1755c_1–61_:GFP proteins containing mutations in the P1′ and P2′ positions. Image panels and illustrations are as in Fig. 1. P1′ position mutated to Asp (D); P1′ and P2′ positions are mutated to Tyr–Gly (E) or Asn–Gly (F). G. Localization of PFI1755c_1–61_:GFP with the P2′ position mutated to Arg. In early parasites GFP accumulated within the parasite (upper panel). In late parasites GFP was distributed around the parasite periphery but the fluorescence intensity was low (lower panel). H. Quantification of GFP export in parasites expressing PFI1755c_1–61_:GFP with either wild-type or mutated HT motifs. The percentage of GFP fluorescence localized in the erythrocyte cytoplasm is shown. The GFP-tagged proteins expressed in each parasite are indicated. Error bars show standard deviation.

Mutation of the P2′ position in PFI1755c_1–61_:GFP from Glu to Ala did not block protein export (Fig. 2B and H) and did not significantly reduce the efficiency of plasmepsin V processing (Fig. 2C, closed arrow, lane 2). Mutation of the P1′ position of the HT motif in PFI1755c to Asp blocked protein export and led to protein accumulation in the PV (Figs 2D, H and S1D). Plasmepsin V processing was not affected by this mutation as high molecular weight unprocessed protein was not detected (Fig. 2C, lane 5). Tryptic peptides from PFI1755c_1–61_:GFP with the P1′ position mutated to Asp were analysed by mass spectrometry. The most N-terminal peptide identified was DEPVVEEQDLK(K), indicating that the protein was correctly processed by plasmepsin V. Both N-acetylated and non-N-acetylated peptides were identified (Table 1).

We also tested a double mutant in which the P1′ and P2′ positions were mutated to Tyr and Gly. This mimics the P1′ and P2′ amino acids in the HT motif of RESA. Although RESA is exported via a dense granule pathway in early parasites, it is not exported when expressed from a trophozoite stage promoter (Rug *et al*., 2004). In the context of PFI1755c_1–61_:GFP, expressed from the calmodulin promoter, this mutation indeed blocked protein export (Fig. 2E and H), leading to an accumulation of GFP in the PV (Fig. S1E). No high molecular weight bands were detected by Western blotting suggesting that this mutation did not affect processing by plasmepsin V (Fig. 2C, lane 8). Tryptic peptides, derived from PFI1755c_1–61_:GFP with the P1′ and P2′ positions mutated to Tyr–Gly, were analysed by mass spectrometry. The most N-terminal peptide identified corresponded to the sequence YGPVVEEQDSK(K). In this case, only non-N-acetylated peptide was identified. As with other proteins analysed the N-terminus was not entirely homogeneous (Table 1). An efficient block in protein export was also observed if the P1′ and P2′ positions were mutated to Asn–Gly (Figs 2F, H and S1F). This protein was processed by plasmepsin V, as judged by Western blotting (Fig. 2C, closed arrow, lane 7).

Although mutation of the P2′ position to Ala did not affect plasmepsin V processing, mutation to Arg did reduce plasmepsin V cleavage efficiency as several higher molecular weight bands were observed by Western blotting of this mutant (Fig. 2C, open arrow, lane 9). In early parasites GFP fluorescence was found within the parasite (Fig. 2G, upper panel), likely in the ER, as seen previously for HT motif cleavage mutants (Boddey *et al*., 2009). In later parasites, GFP accumulated in the parasite periphery (Fig. 2G, lower panel). Exported GFP was not detectable but fluorescence levels were very low in late parasites.

In summary, we have shown that single mutation of the P1′ residue to Asp, and double mutation of the P1′ P2′ residues to Tyr–Gly or Asn–Gly, blocked protein export at a step downstream of plasmepsin V processing. Furthermore, we have shown that the P2′ position can influence cleavage of the HT motif by plasmepsin V. Together these data show that the P1′ and P2′ residues are necessary for efficient protein export.

### The P1′ and P2′ positions in the HT motifs of KAHRP and PfEMP3 are important for efficient protein export

**Fig. 3:**
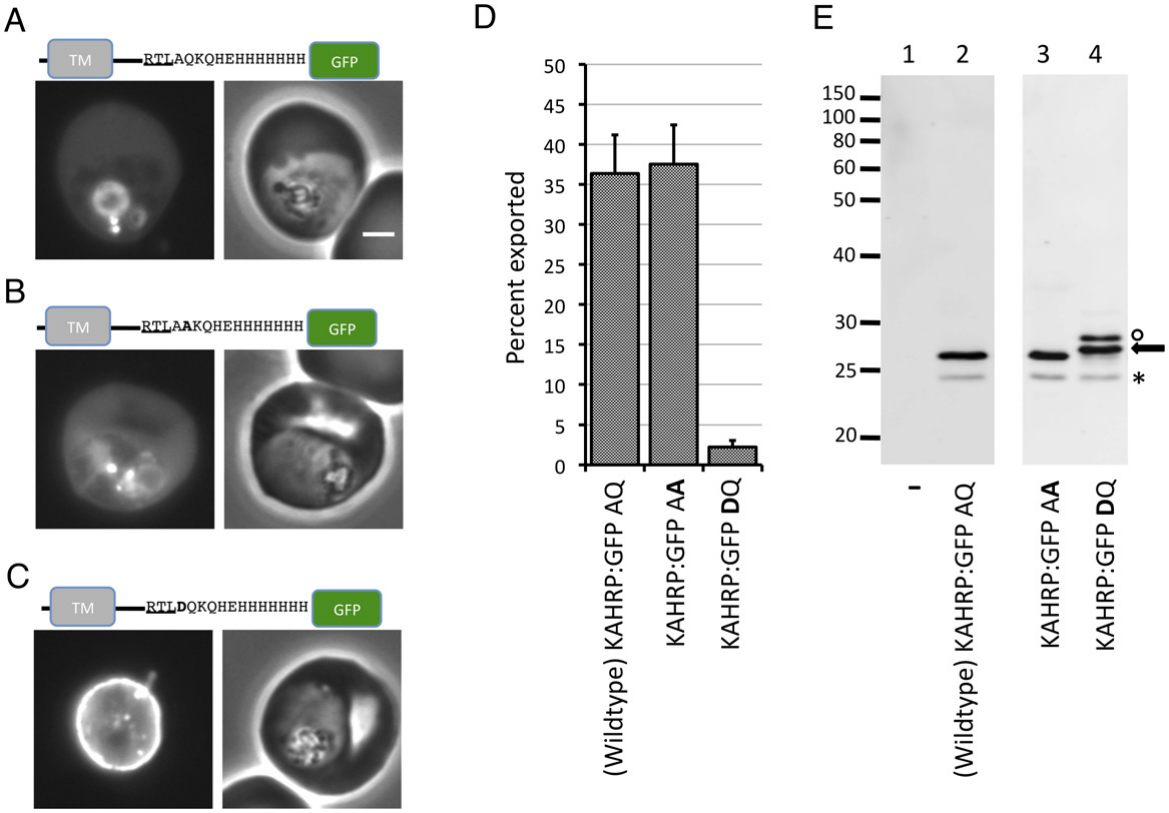
Mutations in the P1′ and P2′ positions of GFP-tagged KAHRP block protein export. A–C. Localization of KAHRP_1–69_:GFP proteins containing mutations in the P1′ and P2′ positions. Image panels and illustrations are as in Fig. 1. Scale bar, 2 μm. Wild-type KAHRP_1–69_:GFP (A); P2′ position mutated from Gln to Ala (B); P1′ position mutated to Asp (C). D. Quantification of GFP export in parasites expressing KAHRP_1–69_:GFP with either wild-type or mutated HT motifs. The percentage of GFP fluorescence localized in the erythrocyte cytoplasm is shown. The GFP-tagged protein expressed in each parasite is indicated. Error bars show standard deviation. E. Anti-GFP Western blot of KAHRP_1–69_:GFP containing mutations in the P1′ and P2′ positions. Below the blot, the construct type is indicated and followed by the residues in the P1′ and P2′ positions; mutated residues are in bold. Open circle: alternately processed form of the protein. Closed arrow: protein that has been processed by plasmepsin V. Asterisk: GFP degradation product. Lane 1 contains material from untransfected parasites.

We subsequently tested the effects of P1′ and P2′ mutations in other model proteins. Specifically, we tested whether the P1′ and P2′ residues in the HT motifs of the exported proteins, Knob-Associated Histidine Rich Protein (KAHRP) and PfEMP3, were also important for protein export. KAHRP has the HT motif ^54^RTLAQ^58^ and as shown previously (Knuepfer *et al*., 2005a), a chimera of residues 1–69 fused to GFP was efficiently exported (Fig. 3A). Mutation of the KAHRP P2′ Gln to Ala had no effect on protein export (Fig. 3B and D). Proteolytic processing of this mutant by plasmepsin V was similar to that of the wild-type protein (Fig. 3E, closed arrow, compare lanes 2 and 3). When the P1′ Ala residue was mutated to Asp, GFP accumulated in the PV (Figs 3C, D and S2C). Western blotting of this mutant revealed protein that had been correctly processed by plasmepsin V but an additional higher molecular weight band was also observed (Fig. 3E, lane 4, open circle). The mass of this band appears smaller than would be expected for the unprocessed protein. Therefore, we postulate that it may be the result of a cleavage event at an alternate site. Nonetheless, the effects of these P1′ mutations are consistent with our observations for equivalent mutations in the HT motif of PFI1755c.

**Fig. 4:**
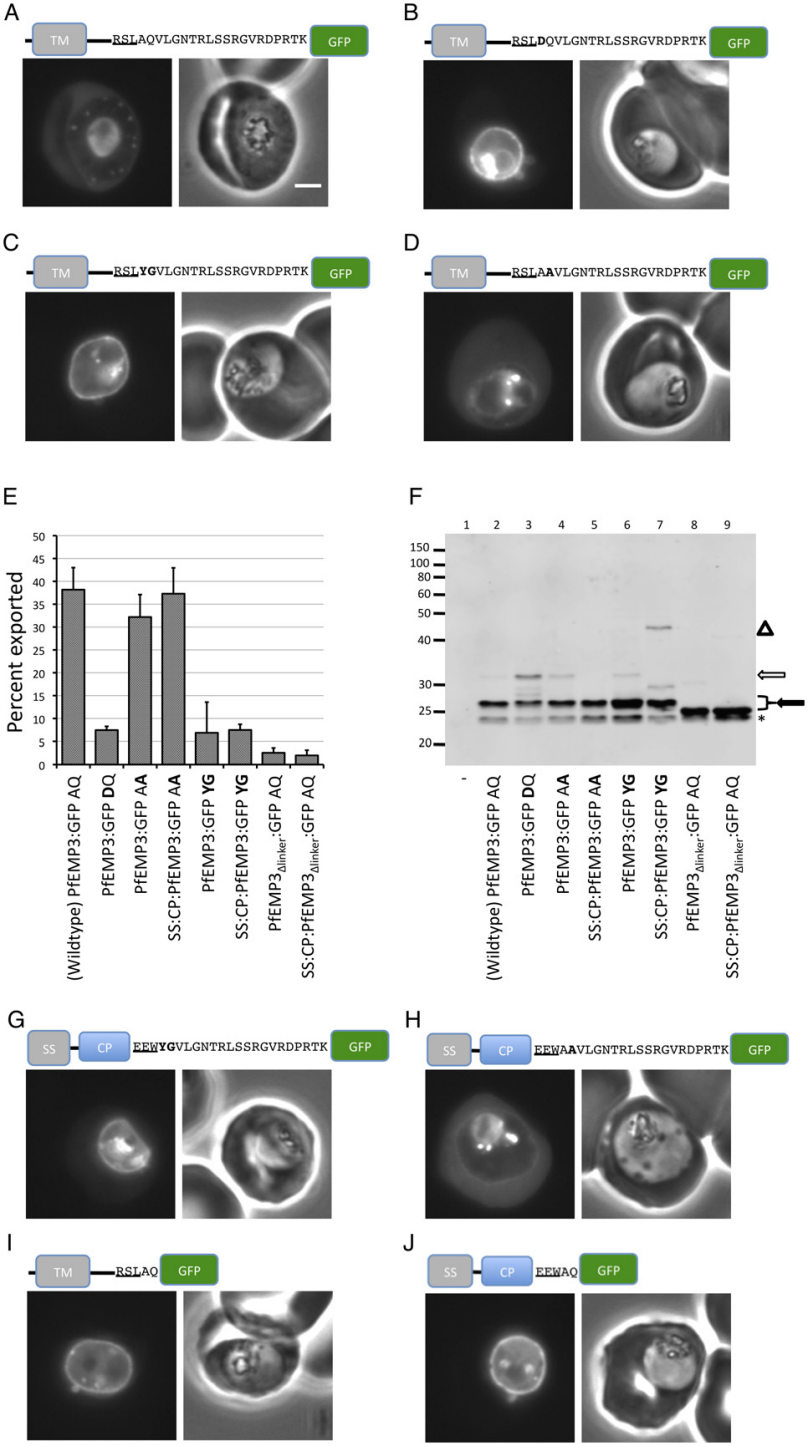
Mutations in the P1′ and P2′ position of PfEMP3 block protein export. A–D. Localization of PfEMP3_1–82_:GFP proteins containing mutations in the P1′ and P2′ positions. Image panels and illustrations are as in Fig. 1. Scale bar, 2 μm. Wild-type PfEMP3_1–82_:GFP (A); P1′ position mutated to Asp (B); P1′ and P2′ residues mutated to Tyr–Gly (C); P2′ position mutated to Ala (D). E. Quantification of GFP export in parasites expressing PfEMP3_1–82_:GFP or SS:CapProt:PfEMP3_63–82_:GFP proteins. The percentage of GFP fluorescence localized in the erythrocyte cytoplasm is shown. The GFP-tagged proteins expressed in each parasite are indicated. Error bars show standard deviation. F. Anti-GFP Western blot of GFP-tagged PfEMP3 proteins, annotated as in Fig. 3E. Triangle: SS:CapProt:PfEMP3_63–82_:GFP that has not been processed by the capsid protease. Open arrow: high molecular weight protein bands that have not been processed by plasmepsin V. Closed arrow: protein that has been processed by either plasmepsin V or capsid protease. Asterisk: GFP degradation product. Lane 1 contains material from untransfected parasites. G–H. Localization of SS:CapProt:PfEMP3_63–82_:GFP proteins containing mutations in the P1′ or both the P1′ and P2′ positions. Image panels and illustrations are as in Fig. 1. P1′ and P2′ residues mutated to Tyr–Gly (G); P1′ residue mutated to Ala (H). I–J. Localization of PfEMP3 constructs in which the linker following the HT motif is deleted. PfEMP3_63–64_:GFP (I); SS:CapProt:PfEMP3_63–64_:GFP (J).

Mutations were also introduced into the P1′ and P2′ positions of PfEMP3_1–82_:GFP. PfEMP3_1–82_:GFP was efficiently exported into the host erythrocyte (Fig. 4A). Mutation of the P1′ Ala to Asp (Figs 4B, E and S3C) or mutation of the P1′ and P2′ positions to Tyr–Gly (Figs 4C, E and S3D) led to GFP being predominantly localized in the PV. As seen with other exported GFP proteins, PfEMP3_1–82_:GFP with the P2′ Gln mutated to Ala was efficiently exported to the host erythrocyte cytosol (Fig. 4D and E).

Some accumulation of high molecular weight bands, consistent with reduced plasmepsin V processing, was seen in Western blots of several PfEMP3 mutants, most notably when the P1′ amino acid is mutated to Asp (Fig. 4F, open arrow, lane 3). However, in all cases the predominant protein species appeared to be correctly processed.

Our analyses show that the effects of mutations of the P1′ and P2′ residues are consistent across a range of model proteins. Together with our experiments using PFI1755c, these experiments also show that the identity of the amino acids in the P1′ and P2′ positions is important for a key targeting step which is likely downstream of plasmepsin V processing.

### Proteins with N-terminal sequences generated by plasmepsin V or capsid protease cleavage are exported via the same pathway

In the following experiments, P1′ and P2′, mutations were introduced into model proteins in which the N-terminus of an exported protein was generated by capsid protease cleavage. First, this allowed us to establish whether the capsid protease-based model proteins and exported proteins processed by plasmepsin V were similarly affected by P1′ and P2′ mutations and hence, exported via the same export pathway. This is a key conclusion as it is possible that the capsid protease-based constructs are exported via a different export pathway from that used by proteins processed by plasmepsin V. Only if the capsid protease-based constructs are exported by the same export pathway as proteins processed by plasmepsin V can we conclude that the cleaved C-terminal fragment of the HT motif is sufficient to mediate export of HT motif-containing proteins. Second, as the capsid protease-based proteins are not processed by plasmepsin V, we were able to clearly establish whether the amino acids in the P1′ and P2′ positions in the HT motif are important for steps in the export pathway that occur downstream of plasmepsin V cleavage.

**Fig. 5:**
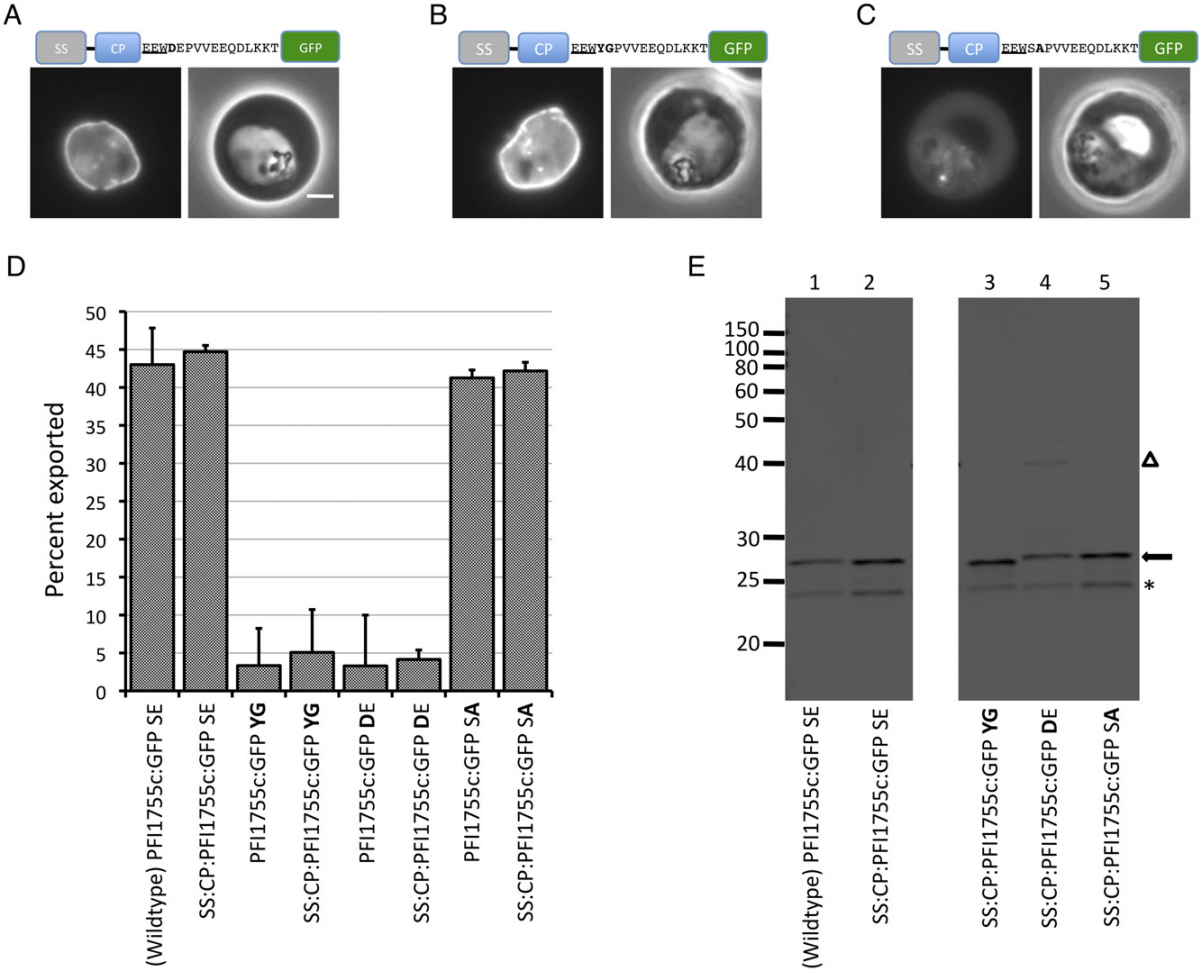
Block in export by mutations in the P1′ and P2′ positions of proteins cleaved by the capsid protease. A–C. Localization SS:CapProt:PFI1755c_49–61_:GFP containing mutations in the P1′ and P2′ positions. Image panels and illustrations are as in Fig. 1. Scale bar, 2 μm. P1′ position mutated to Asp (A); P1′ and P2′ positions mutated to Tyr–Gly (B); P2′ position is mutated to Ala (C). D. Quantification of GFP export in parasites expressing wild-type and mutated versions of PFI1755c_1–61_:GFP or SS:CapProt:PFI1755c_49–61_:GFP. The percentage of GFP fluorescence localized in the erythrocyte cytoplasm is shown. The GFP-tagged proteins expressed in each parasite are indicated (for comparison the efficiency of protein export of similar mutations introduced into PFI1755c_1–61_:GFP are also shown, as in Figs 1 and 2). Error bars show standard deviation. E. Anti-GFP Western blot of parasites expressing PFI1755c_1–61_:GFP or SS:CapProt:PFI1755c_49–61_:GFP and similar constructs in which the P1′ and P2′ residues are mutated. The constructs, followed by the residues in the P1′ and P2′ positions, are indicated below the blot; mutated residues are in bold. Triangle: SS:CapProt:PFI1755c_49–61_:GFP that has not been processed by the capsid protease. Closed arrow: protein that has been processed by either plasmepsin V or capsid protease. Asterisk: GFP degradation product.

Mutations were introduced into the P1′ and P2′ positions in the context of SS:CapProt:PFI1755c_49–61_:GFP. Mutation of the P1′ Ser of SS:CapProt:PFI1755c_49–61_:GFP to Asp efficiently blocked export of this construct leading to an accumulation of GFP in the PV (Figs 5A and S1G). Likewise, mutation of the P1′ P2′ sequence to Tyr–Gly, also blocked protein export (Fig. 5B) leading to PV accumulation of GFP (Fig. S1H). Quantification of the block in export that occurred for each of these mutations shows that they behave similarly in proteins processed by both capsid protease and plasmepsin V (Fig. 5D). Western blotting of the proteins indicated that they were correctly processed and cleaved efficiently by the capsid protease (Fig. 5E, closed arrow, P1′ P2′ Tyr–Gly, lane 3; P1′ Asp, lane 4); only weak bands corresponding to uncleaved protein were observed (Fig. 5E, triangle). When the P2′ Glu was mutated to Ala in the context of SS:CapProt:PFI1755c_49–61_:GFP, the protein was efficiently exported (Fig. 5C and D). The protein appeared to be correctly processed as assessed by Western blotting (Fig. 5E, closed arrow, lane 5). We performed mass spectrometry analysis of tryptic peptides derived from SS:CapProt:PFI1755c_49–61_:GFP in which the P1′ position was mutated to Asp, or the P1′ and P2′ positions were mutated to Tyr–Gly. As with the equivalent mutations in the context of PFI1755c_49–61_:GFP, the most N-terminal peptides corresponded to DEPVVEEQDLK(K) and YGPVVEEQDSK(K) respectively (Table 1). N-acetylated and non-N-acetylated versions of the DEPVVEEQDLK(K) peptide were identified but only the non-acetylated YGPVVEEQDSK(K) peptide was detected. Similarly, analysis of the SS:CapProt:PFI1755c_49–61_:GFP with the P2′ position mutated to Ala revealed that the most N-terminal peptide corresponded to the peptide N-acetyl-SAPVVEEQDLK(K). Some heterogeneity of the N-terminal sequences was seen particularly when the P1′ and P2′ residues were mutated to Tyr–Gly (Table 1).

We also introduced mutations that blocked export of PfEMP3_1–82_:GFP into SS:CapProt:PfEMP3_63–82_:GFP. An efficient block in export was observed when the P1′ and P2′ residues were mutated to Tyr and Gly respectively (Fig. 4G). The mutant protein accumulated in the PV (Fig. S3E). Quantification of the amount of GFP exported into the erythrocyte cytoplasm showed that this mutation behaved similarly in constructs cleaved by either capsid protease or plasmepsin V (Fig. 4E). Western blotting of SS:CapProt:PfEMP3_63–82_:GFP with the P1′ P2′ Tyr–Gly mutation indicated that the majority of the protein was correctly processed by the capsid protease; only weak bands corresponding to uncleaved protein were observed (Fig. 4F, triangle, lane 7). Robust export of SS:CapProt:PfEMP3_63–82_:GFP was observed when the P2′ Gln was mutated to Ala (Fig. 4H and E); this mutation therefore behaved in a similar manner in substrates cleaved by either capsid protease or plasmepsin V. Western blotting of SS:CapProt:PfEMP3_63–82_:GFP with P2′ Gln mutated to Ala indicated efficient processing of the mutant protein (Fig. 4F, lane 5).

It has previously been shown that close proximity of the HT motif to a tightly folded GFP domain can prevent protein export (Knuepfer *et al*., 2005b). PfEMP3_1–82_:GFP, has an 18 residue linker between the HT motif and the GFP domain. A GFP-tagged PfEMP3 construct was generated that lacked this linker, such that the N-terminal P1′ and P2′ residues (Ala Gln) directly preceded the GFP domain (PfEMP3_1–64_:GFP). This protein contained the wild-type HT motif but was not exported (Fig. 4I), and accumulated in the PV (Fig. S3F). When the same N-terminus was generated in the context of the capsid protease SS:CapProt:PfEMP3_63–64_:GFP, a block in export was also observed (Fig. 4J and E) leading to an accumulation of GFP in the PV (Fig. S3G). Both PfEMP3_1–64_:GFP and SS:CapProt:PfEMP3_63–64_:GFP were proteolytically processed, as assessed by Western blotting (Fig. 4F, lanes 8 and 9 respectively). These results show that the block in export observed as a consequence of proximity of the wild-type HT motif to the GFP domain, occurs when the P1′ P2′ residues are generated by plasmepsin V or the capsid protease.

In summary, diverse mutations in the HT motif P1′ and P2′ positions and the following linker region, behaved in a similar manner in the context of two model proteins processed by either plasmepsin V or by the capsid protease. This shows that whether their new N-termini were generated by plasmepsin V or by the capsid protease, the proteins were trafficked via the same export pathway. Importantly, this confirms that the capsid protease constructs are not exported by a non-HT motif dependent export pathway. Furthermore, this allows us to conclude that following plasmepsin V cleavage, it is the C-terminal portion of the HT motif that mediates protein export.

### The context of the HT motif can influence protein export

The preceding results show that the C-terminal fragment of the cleaved HT motif is both necessary and sufficient to mediate export and that the P1′ and P2′ residues of the cleaved HT motif are important for protein export. We sought to determine whether additional amino acids within the linker region between the HT motif and GFP domain of PFI1755c_1–61_:GFP also influenced protein export. To do this we used a PFI1755c_1–61_:GFP construct that contained the P2′ position mutated from E to A, and introduced multiple mutations into the following linker region. As we show later, this model protein was more sensitive to mutations within the linker region, and hence yielded more robust mutant phenotypes, than the model protein with an E in the P2′ position. These experiments allowed us to show that the linker sequence following the HT motif can affect export efficiency but also that the identity of the P2′ amino acid of the HT motif can be particularly important for efficient export in certain sequence contexts.

**Fig. 6:**
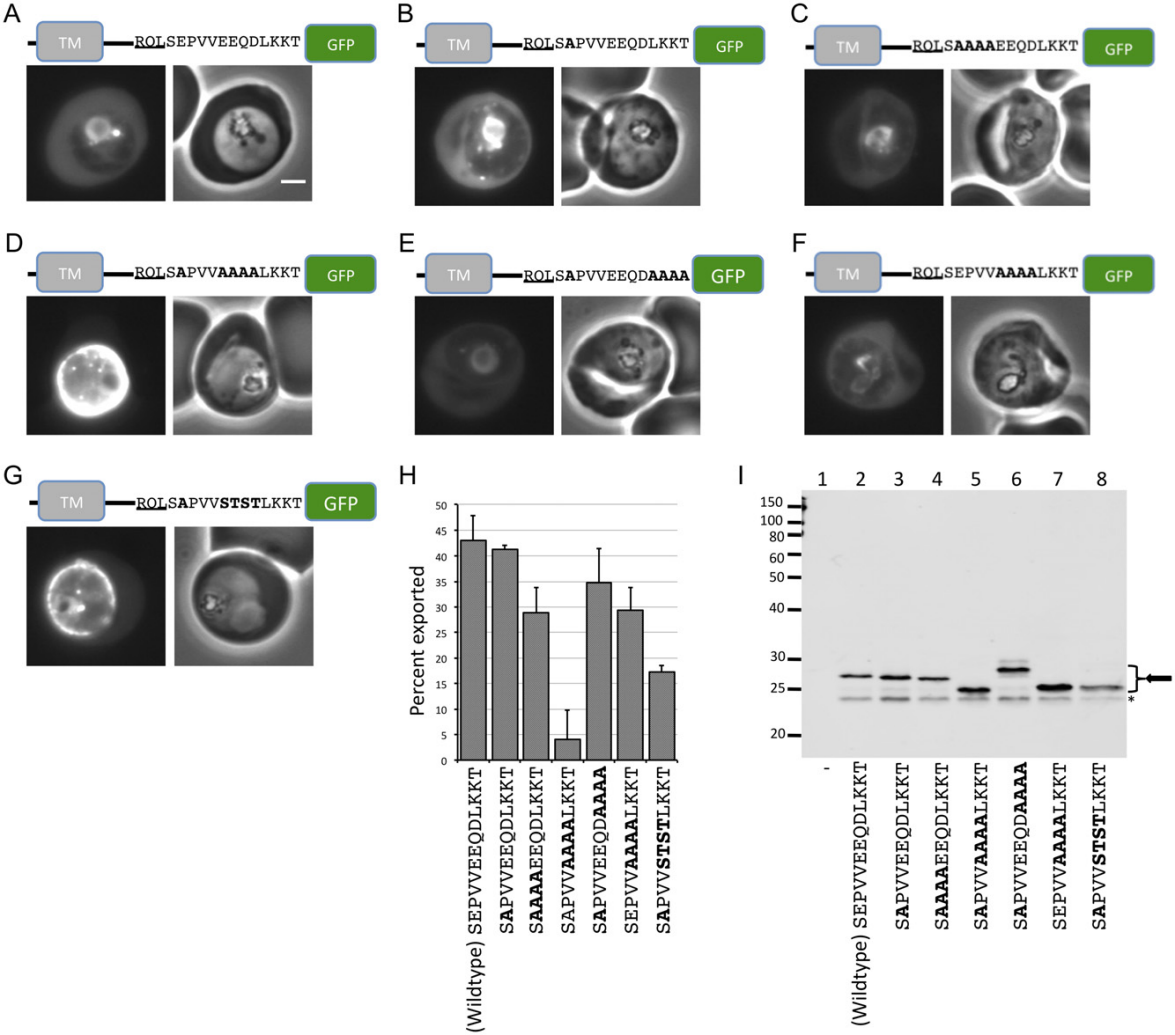
Mutations beyond the P1′ and P2′ positions influence the export efficiency of GFP-tagged PFI1755c. A–G. Images of parasites expressing PFI1755c_1–61_:GFP with mutations in the P2′ position and in the linker sequence. Image panels and illustrations are as in Fig. 1. Scale bar, 2 μm. PFI1755c_1–61_:GFP (A); P2′ position mutated to Ala (B); linker region following the HT motif cleavage site mutated to S**AAAA**EEQDLKKT (C), S**A**PVV**AAAA**LKKT (D), S**A**PVVEEQD**AAAA** (E); SEPVV**AAAA**LKKT (F) or S**A**PVV**STST**LKKT (G). H. Quantification of GFP export in parasites expressing PFI1755c_1–61_:GFP containing mutations in the P2′ residue and following sequence. The percentage of GFP fluorescence localized in the erythrocyte cytoplasm is shown. The GFP-tagged proteins expressed in each parasite are indicated. Error bars show standard deviation. I. Anti-GFP Western blot of GFP-tagged PFI1755c_1–61_:GFP protein containing mutations in the P2′ residue and following sequence. For each construct, the protein sequence following the HT motif cleavage site is indicated below the blot. Closed arrows: protein that has been processed by plasmepsin V. Asterisk: GFP degradation product. Lane 1 contains material from untransfected parasites.

In the model protein PFI1755c_1–61_:GFP, mutation of the P2′ Glu to Ala did not affect protein export (Fig. 6B and H). After HT motif cleavage, the N-terminus of PFI1755c_1–61_:GFP with P2′ Glu to Ala has the sequence S**A**PVVEEQDLKKT; the mutated residue is in bold. In the context of this mutant, we mutated multiple residues downstream of the P1′ and P2′ position of the HT motif. Export was not greatly affected in mutants whose N-terminus, after plasmepsin V cleavage, corresponded to the sequence S**AAAA**EEQDLKKT or S**A**PVVEEQD**AAAA** (Fig. 6C and E respectively, and Fig. 6H). However, a PFI1755c_1–61_:GFP protein whose new N-terminus after HT motif processing was S**A**PVV**AAAA**LKKT, was not efficiently exported (Fig. 6D and H) and accumulated in the PV (Fig. S1J). When the same mutation was introduced into a construct with a wild-type HT motif i.e. with the new N-terminal sequence SEPVV**AAAA**LKKT, GFP was robustly exported (Fig. 6F and H). A small amount of GFP was retained within these parasites. However, the robust export of this construct indicates that the EEQD to AAAA mutation alone does not generate a protein refractory to export. We also tested an alternative construct with the linker region S**A**PVV**STST**LKKT, in which these residues were mutated to polar amino acids. In this mutant, a low level of GFP fluorescence was detectable in the host cell cytosol but the protein was predominantly localized in the PV (Figs 6G, H and S1K). When analysed by Western blotting, it was clear that many of the PFI1755c_1–61_:GFP proteins with AAAA mutations within the linker region exhibited altered electrophoretic mobility relative to the wild-type PFI1755c_1–61_:GFP and P2′ Glu to Ala mutants (Fig. 6I, closed arrow).

To determine the identity of the N-terminal residues, we immunoprecipitated proteins with the linker sequences S**A**PVV**AAAA**LKKT and S**A**PVV**STST**LKKT and analysed peptides using mass spectrometry. For the PFI1755c_1–61_:GFP with the linker sequence S**A**PVV**AAAA**LKKT, the most N-terminal peptide detected had the sequence S**A**PVV**AAAA**LK(K). Only N-acetylated peptides were identified (Table 1). The most N-terminal peptides identified for PFI1755c_1–61_:GFP with the linker region S**A**PVV**STST**LKKT started with the sequence S**A**PVV**STST**LK(K). Again, only N-acetylated peptides were identified (Table 1). These data confirm that in both cases, HT motif cleavage yielded proteins with the expected N-termini.

These data show that the amino acid sequence downstream of the HT motif can influence protein export and that the importance of the amino acid in the P2′ position of the HT motif is dependant upon the downstream sequence. As the protein with the new N-terminus SAPVVAAAALKKT is processed by plasmepsin V and N-acetylated we also conclude that neither plasmepsin V cleavage *per se*, nor an N-acetylated N-terminus is sufficient for efficient protein export.

## Discussion

The malaria parasite exports many proteins into its host erythrocyte. The majority of exported proteins contain a HT motif that has the consensus RxLxE/D/Q, which is cleaved in the ER by the protease plasmepsin V. A number of models have been proposed to explain the contribution of the HT motif to protein export. The only mutations shown to completely block protein export are in the first and third positions of the HT motif (Arg and Leu respectively) and block processing by plasmepsin V.

We show that the C-terminal portion of the cleaved HT motif is sufficient to mediate protein export. Proteins lacking a HT motif but whose new N-termini resemble the cleaved HT motif, generated by the capsid protease, not plasmepsin V, were efficiently exported. Our results differ from previous work showing that a protein whose N-terminus resembled the cleaved HT motif was not exported (Boddey *et al*., 2010). In the latter experiments, signal peptidase was used to generate a protein whose N-terminus resembled the cleaved HT motif. In the context of these experiments it is likely that the capsid protease generates the N-terminus with greater accuracy than signal peptidase. The consensus sequence for signal peptidase cleavage is uncomplicated and cleavage may occur at multiple sites proximal to the signal peptide (Kotia and Raghani, #b^1001^). By using an exogenous protease we have bypassed the requirement for plasmepsin V cleavage. This shows that plasmepsin V cleavage is not directly coupled to the hand-off of a protein to the next step in the export pathway as proposed previously (Boddey *et al*., 2010). Rather, our data support a model in which the role of plasmepsin V is to cleave off the N-terminal transmembrane domain and generate a new N-terminal sequence that is sufficient to function as a signal for protein export. Notably, the ability of this sequence to mediate export is blocked if it is preceded by a folded domain (such as the capsid protease domain with an active site mutation). This suggests that the free N-terminus of this sequence is also an important feature of the signal for export.

Our exported proteins whose N-terminal export sequences were generated by the capsid protease lacked part of the HT motif (RxL). This region, with some contribution from the P2′ position, has been implicated in PI(3)P-binding in the ER lumen (Bhattacharjee *et al*., 2012b). Robust export of proteins whose N-terminal export sequences were generated by the capsid protease suggests that recognition of the new N-terminal export sequence by putative export machinery may occur downstream of the PI(3)P sorting event.

Mutagenesis of the P1′ P2′ amino acids showed that this region is necessary for protein export. Mutation of the P1′ position to Asp blocked protein export. Furthermore, mutation of the P1′ and P2′ positions to Tyr–Gly or Asn–Gly also blocked export. Likely a number of other double mutations in the P1′ and P2′ positions of the HT motif will also block protein export. Although our experiments clearly show that the identity of the P2′ amino acid is important for export, surprisingly mutation of the P2′ amino acid to Ala alone, did not block protein export; a result also seen by others (Grüring *et al*., 2012; Bhattacharjee *et al*., 2012a). Some studies have seen a decrease in export of reporter proteins after mutation of the P2′ position to Ala (Przyborski *et al*., 2005; Boddey *et al*., 2009). The reason for this discrepancy is unclear; it is possible that the effects of the P2′ Ala mutations are influenced by the sequences that follow the HT motifs of different model proteins. Notably, we show that mutating the PFI1755c P2′ residue to Ala reduces the efficiency of protein export but only when combined with further mutations in the linker region following the HT motif. It would be interesting to investigate the contribution to export of the regions downstream of the HT motifs of other exported proteins. Additionally, expression of model proteins from different promoters might also influence whether mutation of the P2′ position affects export (Boddey *et al*., 2009).

We also showed that in the context of PFI1755c_1–61_:GFP, mutation of the P2′ residue to Arg reduced the efficiency of cleavage of the HT motif by plasmepsin V. This is the first instance where a HT motif residue, other than those in the first and third positions, has been shown to influence processing of the HT motif by plasmepsin V. Overall our data show that the P2′ residue is important for efficient proteolytic processing of the HT motif and that both the P1′ and P2′ residues, upon cleavage of the HT motif, form part of the signal that is recognized in a step downstream of plasmepsin V processing. However, as for the signals that mediate protein secretion by bacterial type III and IV secretion systems, there does not appear to be an extensive and rigid consensus sequence for the export signal (Galán and Wolf-Watz, 2006; Alvarez-Martinez and Christie, 2009). Resident PV proteins might avoid engaging with the export apparatus by having structured N-termini, N-terminal residues that do not favour interaction with the export apparatus, or having a tightly folded structure (Gehde *et al*., 2009).

Importantly, we showed that P1′ and P2′ mutations had the same effect on export of proteins whose N-terminii were generated by plasmepsin V or by the capsid protease. Likewise, in both contexts removing the linker sequence that separates the P1′ and P2′ residues from the folded GFP domain blocked export. These are key observations as they show that proteins whose N-termini are generated by capsid protease, and only contain the C-terminal portion of the HT motif, follow the same export pathway as proteins that are processed by plasmepsin V. This is important as it allows us to conclude that the C-terminal fragment of the HT motif is both necessary and sufficient to mediate the normal export of proteins that contain a HT motif once they are processed by plasmepsin V. While this paper was under review, Grüring and colleagues (2012), using a different approach, published a study that strongly supports this model. These data indicate that the new N-terminus of exported proteins, generated by plasmepsin V cleavage, is a key feature of the export signal in proteins that contain a HT motif.

A linker region between the HT motif and GFP domain is required for protein export (Knuepfer *et al*., 2005a). There is little sequence conservation downstream of the HT motif and the linker sequence can be replaced by a stretch of alanines in the context of a wild-type HT motif. However, we have shown that in the context of a mutation in the P2′ position of the HT motif, mutation of acidic residues in the sequence downstream of the HT motif can influence protein export efficiency. Therefore, in addition to its role in separating the HT motif from the GFP domain of model proteins, the linker region sequence can also contribute to the export signal. It has previously been shown that mutation of charged residues within exported proteins can influence protein export (Spielmann and Gilberger, 2010). This effect may be particularly important for the efficient export of proteins with suboptimal HT motifs.

Our findings also address the relationship between N-acetylation and protein export. As shown previously (Boddey *et al*., 2009) we find that N-acetylation of the N-terminus generated by HT motif cleavage is not sufficient to mediate protein export. The PFI1755c mutant with the cleaved N-terminal sequence S**A**PVV**AAAA**LKKT:GFP was not efficiently exported although it was N-acetylated, localized in the parasite secretory pathway, and had an N-terminal sequence that is of similar length to the N-terminal sequences of exported proteins. It remains unclear whether N-acetylation is required for export. However, we identified non-acetylated peptides from P1′ and P2′ mutants that were significantly blocked in protein export. Identification of the N-acetyltransferase for exported proteins will be crucial to gain a better understanding of the signal that directs proteins for export.

Export of proteins into the infected erythrocyte is essential for parasite survival and virulence. Our data show that cleavage of the HT motif of exported proteins exposes an N-terminal sequence that, although lacking a strong consensus, contains sufficient information to mediate protein export.

## Experimental procedures

### Plasmids

DNA encoding PFI1755c, PfEMP3 and KAHRP was PCR amplified from *P. falciparum* 3D7 cDNA and cloned into a *P. falciparum* expression plasmid, in frame with 3′ GFP. Expression was under the control of pfCAM 5′ and pbDT 3′ regions. The constructs were named PFI1755c_1–61_:GFP (GenBank ID: JX279925), PfEMP3_1–82_:GFP (GenBank ID: JX279927) and KAHRP_1–69_:GFP (GenBank ID: JX279926) respectively. Similar expression plasmids were used to generate SS:CapProt:PFI1755c_49–61_:GFP (GenBank ID: JX279928), and SS:CapProt:PfEMP3_63–82_:GFP (GenBank ID: JX279929). Semliki forest virus capsid protease gene includes the mutation V149S that does not affect protease activity (numbering as in UniProt entry P03315). The active site capsid protease mutant S138A corresponds to residue 219 in the full-length viral protein (Choi *et al*., 1997). DNA fusions and mutations were generated using overlapping PCR.

### Parasite culture and transfection

PFI1755c linker mutants, PFI1755c capsid protease, PFI1755c with P1′ and P2′ amino acids mutated to Asn–Gly, and KAHRP mutants were transfected into 3D7*attB* parasites using pINT (Nkrumah *et al*., 2006) as described previously (Deitsch *et al*., 2001). Other PFI1755c P1′, P2′ mutants and PfEMP3 constructs were transfected into 3D7 parasites using pHTH (Balu *et al*., 2005).

### Microscopy

Parasites were imaged by placing a drop of culture material between a coverslide and coverslip. Fluorescence and phase contrast images were acquired using a Zeiss Axiovert S100 microscope, equipped with a HBO100 lamp, an FDI Photonic Sciences camera, and ImagePro Plus software. Fluorescence images were acquired using identical exposure times; equivalent brightness and contrast settings were applied to images using ImageJ. Parasites were fed one day before imaging. Immunofluorescence imaging was carried out as described in the supplementary information.

Z-stacks of images used for quantification of export efficiency were acquired using a Zeiss Axiovert 200 M microscope and Axiovision software (parasites were randomly selected under phase contrast illumination). Images were deconvolved using Volocity software. Z-stack sum-projections were assembled using ImageJ. Within each projection, the area encompassing parasite and the area encompassing the cytoplasm of the infected erythrocyte, were manually defined. GFP fluorescence intensities of each region were determined using ImageJ. The fraction of protein export was calculated by dividing the amount of GFP fluorescence in the red cell only by the amount of GFP fluorescence in the red cell and the parasite. This fraction is expressed as a percentage. Quantification of export in each parasite line was performed in two independent experiments involving a total of 30–35 randomly selected parasites. The data presented are the combined mean of both experiments and error bars show a combined standard deviation for both experiments.

### Protein immunoprecipitation and Western blotting

For analysis by Western blotting, cells from non-synchronous cultures were lysed in 50 mM Tris pH 8.5, 200 mM NaCl, 2 mM EDTA containing a protease inhibitor cocktail (Roche). Culture volumes were adjusted to obtain approximately equivalent amounts of mid- to late-stage parasites in each sample. Lysates were clarified by centrifugation at 18 500 *g*. Proteins were immunoprecipitated using anti-GFP agarose (MBL), analysed by Western blotting using rabbit anti-GFP antibody (Invitrogen), and a LI-COR Odyssey imager.

### Mass spectrometry

Proteins were immunoprecipitated from 30 ml cultures, processed as above, and SDS-PAGE gels were Coomassie stained. Bands were digested as described previously (Shevchenko *et al*., 2007). Peptides were separated using a nanoACQUITY UPLC system coupled to a Synapt HDMS mass spectrometer (Pringle *et al*., 2007) (Waters Corporation). The mass spectometer was operated in a data-independent mode (LC-MS^E^) of acquisition (Silva *et al*., 2005). Raw data were processed using PLGS v2.5. Identified peptides that were present in two out of three technical replicates, had a mass error of less than 10 ppm, had more than 30% matched product coverage and were present in replicate experiments are listed in Table 1. Detailed methods are described in the supplementary information.
