# Supplementary material for: The C-terminal portion of the cleaved HT motif is necessary and sufficient to mediate export of proteins from the malaria parasite into its host cell
Source: Mol Microbiol. 2013 Jan 21;87(4):835–50. doi: 10.1111/mmi.12133 (PMC3567231; doi:10.1111/mmi.12133)
Supplement: Supplementary file 1 [file mmi0087-0835-SD1.docx]

Supplementary information

**Supplementary methods**

**Immunofluorescence analysis**

Infected red blood cells were adhered to poly-L-lysine coated coverslides, fixed in 4% paraformaldehyde, permeabilised with 0.1% Triton X100, and labelled with anti-Exp2 monoclonal antibody 7.7 ([Fischer et al., 1998](#_ENREF_1)) and Alexa 594 labelled secondary antibody. As seen previously, this method did not preserve the red cell cytoplasm or GFP exported into the red cell ([Tonkin et al., 2004](#_ENREF_2)). Although other fixation methods can preserve the red cell more efficiently ([Tonkin et al., 2004](#_ENREF_2)), these were not used as robust Exp2 labelling was not seen in all cells.

**Mass spectrometry**

Nano-Ultra Performance Liquid Chromatography (nUPLC) seperation of tryptic digests was performed using a 10 kpsi nanoACQUITY UPLC system (Waters Corporation) using a 5 µm SYMMETRY C18, 180 µm x 200 mm trap column and a 1.7 µm BEH130 C18, 75 µm x 250 mm analytical column. A two phase linear gradient was performed where solvent A was 0.1 % formic acid in water and solvent B was 0.1 % formic acid in acetonitrile (J.T.Baker). A linear gradient was then applied at a flow rate of 0.3 µl/min whereby the concentration of solvent B was increased from 3% to 40 % over 29 minutes.

Data were acquired using a Synapt HDMS (Waters Corporation) mass spectrometer tuned to a resolution of 10,000 (full width half height). During analysis, a solution of [Glu1]-fibrinopeptide B at 500 fmol/µl was delivered at a flow rate of 0.3 µl/min via a NanoLockSpray source. This faciltated the post acquisition lockmass correction of data using the monoisotopic mass of the doubly charged precursor of [Glu1]-fibrinopeptide B. The reference sprayer was sampled every 60s.

Mass measurements were made using a data independent mode (LC-MS^E^) of acquisition. Briefly, energy in the collision cell was alternated from low energy (4 eV) to high energy (energy ramp from 15-35 eV) whilst continuously aquiring MS data. Measurements were made over a *m/z* range of 100-2000 Da and the time of flight mass analyser was operated in V mode with a scan time of 1s.

All data processing was carried out using PLGS v2.5 (Waters Corporation). A database comprising protein sequences of all proteins used, including variable N-termini, appended to protein sequences of *Plasmodium falciparum* (PlasmoDB release 8.2) and to the common Repository of Adventitious Proteins sequences, was used for all searches. Trypsin was set as the protease and a maxiumum of two missed cleavages were allowed for the identification of semi-tryptic peptides. Carbamidomethyl-C was specified as a fixed modification whilst oxidation of methionine and N-terminal acetylation were included as variable modifications. Identified peptides that were present in 2 out of 3 technical replicates, had a mass error of less than 10 ppm, had more than 30 % matched product coverage (a peptide for which each amino acid was identified by either a b or a y product ion was assigned a coverage of 100 %) and were present in replicate experiments are reported.

Supplementary figures.

**Figure S1. Accumulation of PFI1755c mutant proteins in the parasitophorous vacuole**

Red blood cells infected with parasites expressing mutant versions of GFP tagged PFI1755c fusion proteins, were immunolabelled with anti-Exp2. GFP fluorescence and Exp2 labeling were detected using fluorescence microscopy. From left to right, panels show GFP fluorescence, anti-Exp2 immunofluorescence, an overlay of the GFP and anti-Exp2 fluorescence, and a phase contrast image. A diagram illustrating the construct type is included above each set of corresponding images.

(A) Untransfected *P. falciparum.*

(B) PFI1755c_1-61_:GFP.

(C) SS:CapProt:PFI1755c_49-61_:GFP with S138A mutation.

(D) PFI1755c_1-61_:GFP with the P1’ position mutated to Asp.
(E) PFI1755c_1-61_:GFP with the P1’ and P2’ positions mutated to Tyr Gly; (F) Asn Gly.
(G) SS:CapProt:PFI1755c_49-61_:GFP with the P1’ position mutated to Asp.
(H) SS:CapProt:PFI1755c_49-61_:GFP with the P1’ and P2’ positions mutated to Tyr Gly.

(I) PFI1755c_1-61_:GFP with the P2’ position mutated to Ala.
(J) PFI1755c_1-61_:GFP with the linker sequence SAPVVAAAALKKT; (K) SAPVVSTSTLKKT.
Scale bar, 2µm.

**Figure S2. Accumulation of KAHRP mutant proteins in the parasitophorous vacuole**

Red blood cells infected with parasites expressing KAHRP_1-69_:GFP mutants were immunolabelled with anti-Exp2. GFP fluorescence and Exp2 labeling were detected using fluorescence microscopy. Image panels are as for Figure S1. A diagram illustrating the construct type is included above each set of corresponding images.

(A) Untransfected *P. falciparum.*

(B) KAHRP_1-69_:GFP.

(C) KAHRP_1-69_:GFP with the P1’ position mutated to Asp.

Scale bar, 2µm.

**Figure S3. Accumulation of PfEMP3 mutant proteins in the parasitophorous vacuole**

Red blood cells infected with parasites expressing GFP tagged PfEMP3 mutants, were immunolabelled with anti-Exp2. GFP fluorescence and Exp2 labeling were detected using fluorescence microscopy. Image panels are as for Figure S1. A diagram illustrating the construct type is included above each set of corresponding images.

(A) Untransfected *P. falciparum.*

(B) PfEMP3_1-82_:GFP.

(C) PfEMP3_1-82_:GFP with the P1’ position mutated to Asp.

(D) PfEMP3_1-82_:GFP with the P1’ and P2’ positions mutated to Tyr Gly.

(E) SS:CapProt:PfEMP3_63-82_:GFP with the P1’ and P2’ positions mutated to Tyr Gly.

(F) PfEMP3_1-64_:GFP.

(G) SS:CapProt:PfEMP3_63-64_:GFP.

Scale bar, 2µm.

**Figure S4. Averaged tandem mass spectrum for peptides SAPVVSTSTLK and SEPVVEEQDLKK (derived from SS:CapProt:PFI1755c_49-61_:GFP)**

Mean b and y ion intensities for two experiments, coloured blue and red. Standard deviation of triplicate mesurements for each ion intensity, normalised to the most abundant ion, is shown. ^*^, neutral loss of ammonia; ^o^, neutral loss of water

**References**

Fischer, K., T. Marti, B. Rick, D. Johnson, J. Benting, S. Baumeister, C. Helmbrecht, M. Lanzer & K. Lingelbach, (1998) Characterization and cloning of the gene encoding the vacuolar membrane protein EXP-2 from Plasmodium falciparum. *Molecular and Biochemical Parasitology* **92**: 47-57.

Tonkin, C. J., G. G. van Dooren, T. P. Spurck, N. S. Struck, R. T. Good, E. Handman, A. F. Cowman & G. I. McFadden, (2004) Localization of organellar proteins in *Plasmodium falciparum* using a novel set of transfection vectors and a new immunofluorescence fixation method. *Molecular and Biochemical Parasitology* **137**: 13-21.
