# Supplementary figures and images for: The C-terminal portion of the cleaved HT motif is necessary and sufficient to mediate export of proteins from the malaria parasite into its host cell
Source: Mol Microbiol. 2013 Jan 21;87(4):835–50. doi: 10.1111/mmi.12133 (PMC3567231; doi:10.1111/mmi.12133)

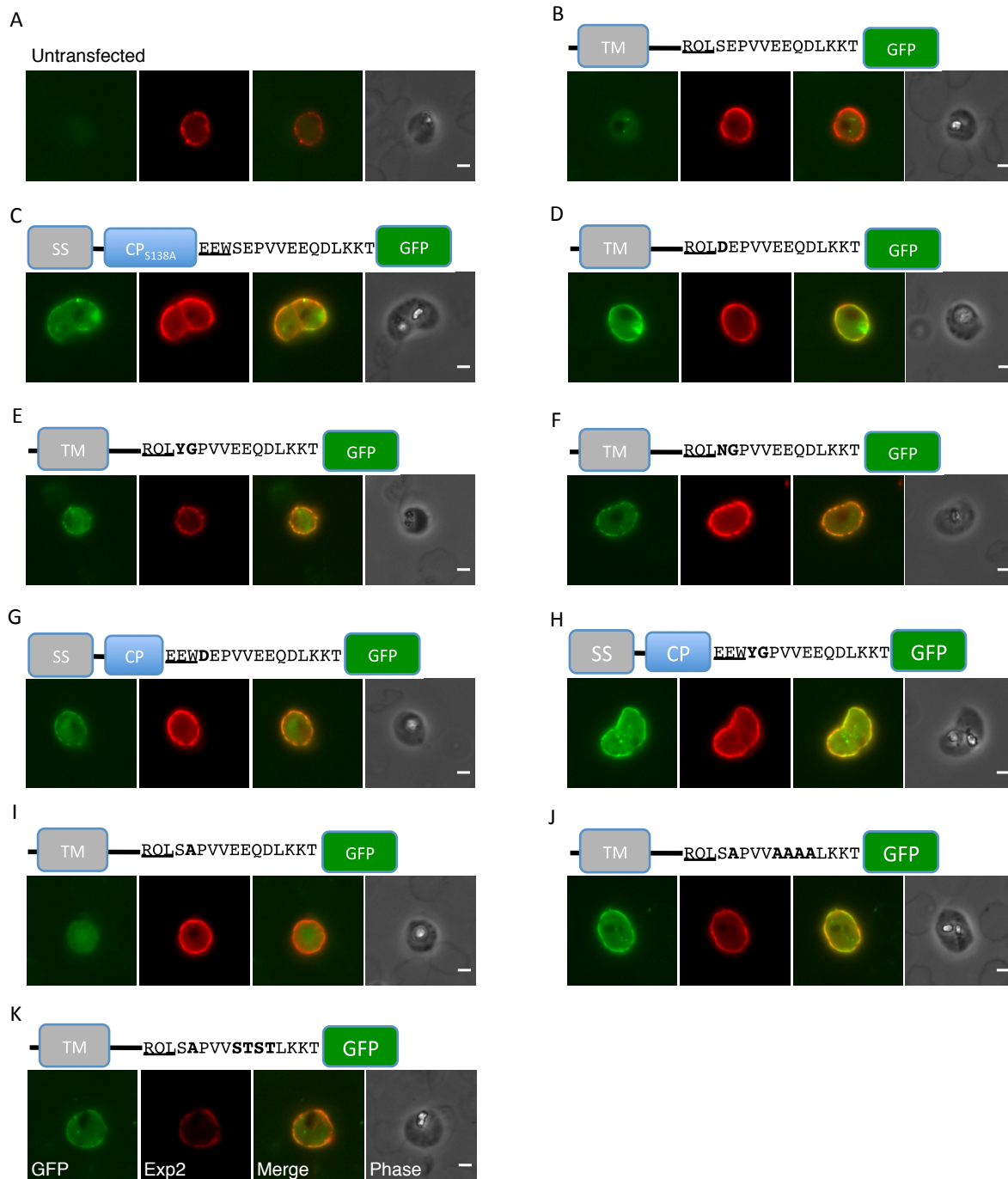

Tarr et al. Supplementary Figure 1

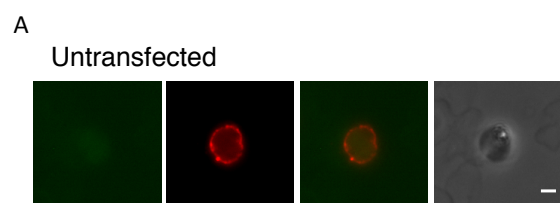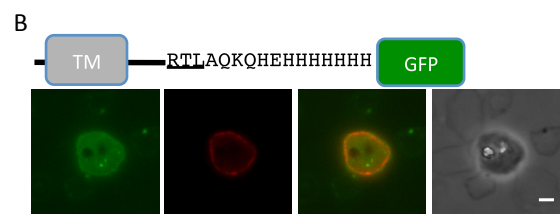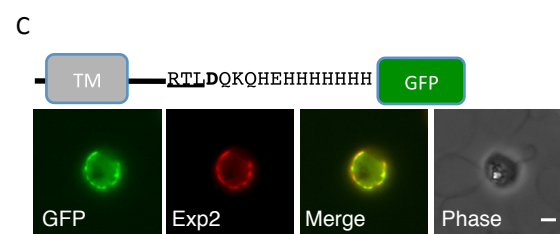

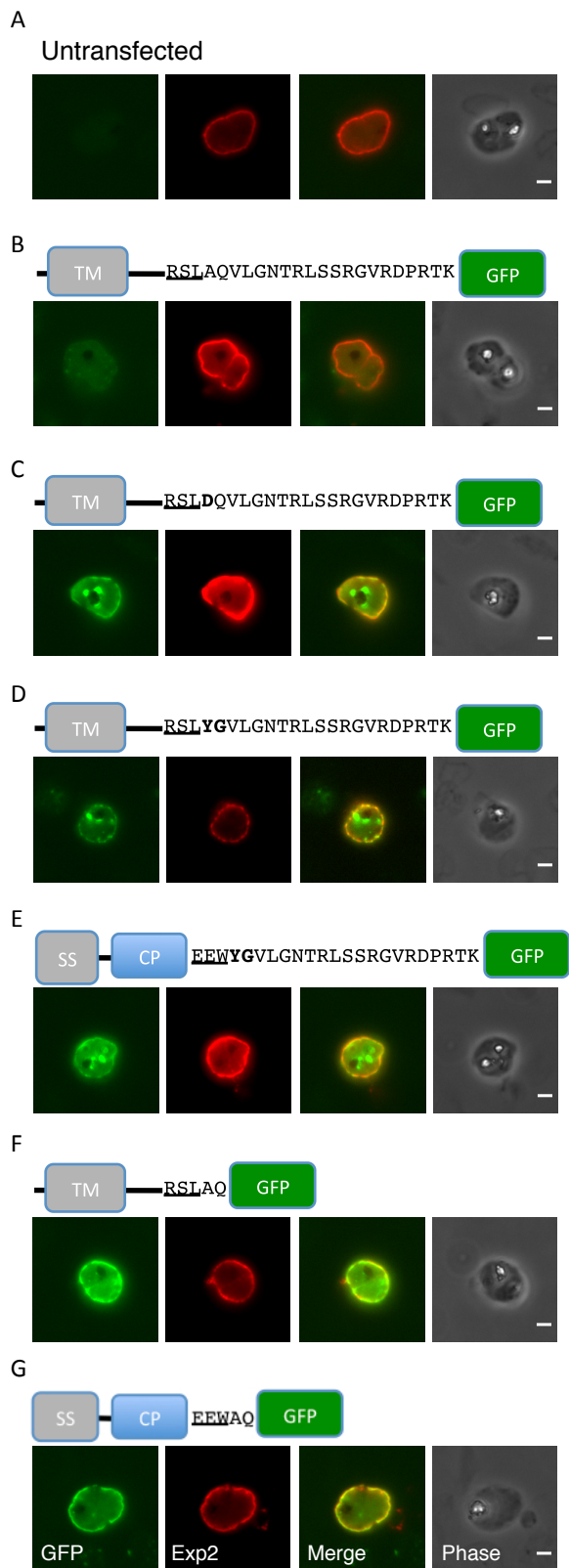

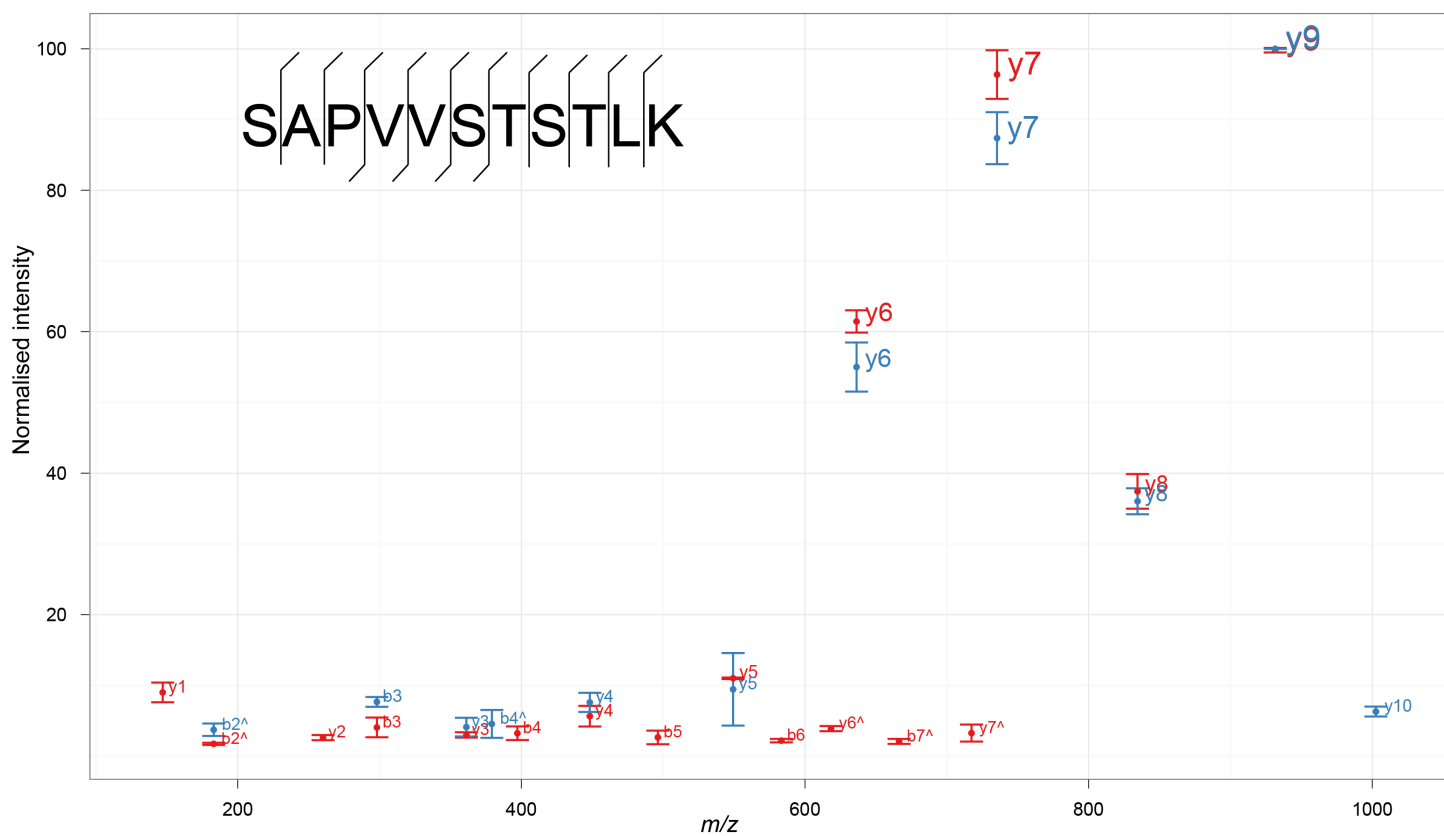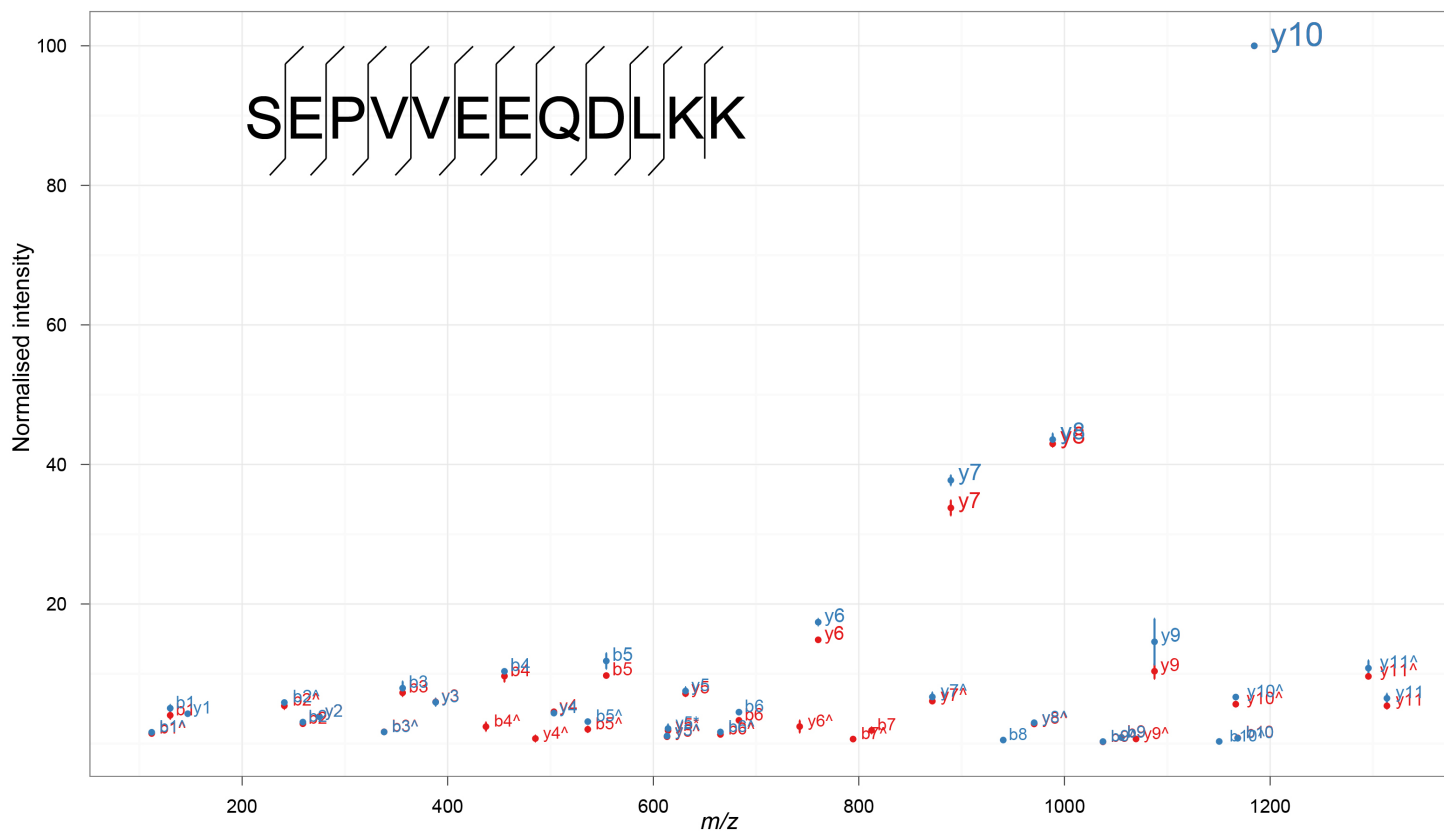

Supplement: Supplementary file 2 [file mmi0087-0835-SD2.pdf]
